# On (Δ^*m*^, *I*)-Statistical Convergence of Order *α*

**DOI:** 10.1155/2014/535419

**Published:** 2014-02-09

**Authors:** Mikail Et, Abdullah Alotaibi, S. A. Mohiuddine

**Affiliations:** ^1^Department of Mathematics, Fırat University, 23119 Elazıg, Turkey; ^2^Department of Mathematics, Faculty of Science, King Abdulaziz University, P.O. Box 80203, Jeddah 21589, Saudi Arabia

## Abstract

The idea of *I*-convergence of real sequences was introduced by Kostyrko et al., (2000/01) and also independently by Nuray and Ruckle (2000). In this paper, we introduce the concepts of (Δ^*m*^, *I*)-statistical convergence of order *α* and strong (Δ_*p*_^*m*^, *I*)-Cesàro summability of order *α* of real sequences and investigated their relationship.

## 1. Introduction

The idea of statistical convergence was given by Zygmund [1] in the first edition of his monograph published in Warsaw in 1935. The concept of statistical convergence was introduced by Steinhaus [2] and Fast [3] and later reintroduced by Schoenberg [4] independently. Later on it was further investigated from the sequence space point of view and linked with summability theory by Connor [5], Çınar et al. [6], Et et al. [7], Fridy [8], Güngör et al. [9, 10], Işik [11], Mohiuddine et al. [12–14], Mursaleen [15], Šalát [16], and many others.

The idea of *I*-convergence of real sequences was introduced by Kostyrko et al. [17] and also independently by Nuray and Ruckle [18] (who called it generalized statistical convergence) as a generalization of statistical convergence. Later *I*-convergence was studied by Das et al. [19–21], Kostyrko et al. [22], Mohiuddine et al. [23–25], Šalát et al. [26, 27], Tripathy and Hazarika [28, 29], and many others.

In this paper, we introduce the concepts of (Δ^*m*^, *I*)-statistical convergence of order *α* and strong (Δ_*p*_^*m*^, *I*)-Cesàro summability of order *α* of real sequences and investigated their relationship. In Section 2 we give a brief overview about statistical convergence, strong *p*-Cesàro summability, *I*-convergence, and difference sequences. In Theorem 8, we give the inclusion relations between the sets of *S* ^*α*^(Δ^*m*^, *I*)-convergent sequences and *w* ^*α*^(Δ_*p*_^*m*^, *I*)-summable sequences. In Theorem 10, we give the relationship between *w* ^*α*^(Δ_*p*_^*m*^, *I*)-summable sequences for different *α*'s. In Theorem 12, we give the relationship between the sets of *S* ^*α*^(Δ^*m*^, *I*)-convergent sequences for different *α*'s.

## 2. Definition and Preliminaries

Let *w* be the set of all sequences of real or complex numbers and let *ℓ*_*∞*_, *c*, and *c*_0_ be, respectively, the Banach spaces of bounded, convergent, and null sequences *x* = (*x*_*k*_) with the usual norm ||*x*|| = sup⁡|*x*_*k*_|, where *k* ∈ *ℕ* = {1,2,…}, the set of positive integers. Also by *bs*, *cs*, *ℓ*_1_, and *ℓ*_*p*_, we denote the spaces of all bounded, convergent, and absolutely and *p*-absolutely convergent series, respectively.

The definitions of statistical convergence and strong *p*-Cesàro convergence of a sequence of real numbers were introduced in the literature independently of one another and followed different lines of development since their first appearance. It turns out, however, that the two definitions can be simply related to one another in general and are equivalent for bounded sequences. The idea of statistical convergence depends on the density of subsets of the set *ℕ*. The density of a subset *E* of *ℕ* is defined by

(1) $$\begin{array}{c} \delta \left(E\right)=\underset{n\to \infty }{\lim}\frac{1}{n}\sum_{k=1}^{n}\chi_{E}\left(k\right)\;\text{provided}\;\text{the}\;\text{limit}\;\text{exists}, \end{array}$$

where *χ*_*E*_ is the characteristic function of *E*. It is clear that any finite subset of *ℕ* has zero natural density and *δ*(*E* ^*c*^) = 1 − *δ*(*E*).

The order of statistical convergence of a sequence of numbers was given by Gadjiev and Orhan in [30] and then statistical convergence of order *α* and strong *p*-Cesàro summability of order *α* were studied by Çolak [31].

The notion of difference sequence spaces was introduced by Kızmaz [32] and it was generalized by Et et al. [33–35] such as

(2) $$\begin{array}{c} \Delta^{m}\left(X\right)=\left\{x=\left(x_{k}\right):\left(\Delta^{m}x_{k}\right)\in X\right\}, \end{array}$$

for *X* = *ℓ*_*∞*_, *c*, or *c*_0_, where *m* ∈ *ℕ*, Δ^0^ *x* = (*x*_*k*_), Δ^*m*^ *x* = (Δ^*m*−1^ *x*_*k*_ − Δ^*m*−1^ *x*_*k*+1_), and so $\Delta^{m}x_{k}=\sum_{i=0}^{m}{\left(-1\right)}^{i}\left(\begin{array}{c} m\\ i \end{array}\right)x_{k+i}.$ The sequence spaces Δ^*m*^(*X*) are Banach spaces normed by

(3) $$\begin{array}{c} {||x||}_{\Delta }=\sum_{i=1}^{m}\left|x_{i}\right|+{||\Delta^{m}x_{k}||}_{\infty }, \end{array}$$

for *X* = *ℓ*_*∞*_, *c*, or *c*_0_. Let *X* be any sequence spaces, if *x* ∈ Δ^*m*^(*X*), then there exists one and only one *y* = (*y*_*k*_) ∈ *X* such that

(4) xk=∑i=1k−m(−1)m(k−i−1m−1)yi=∑i=1k(−1)m(k+m−i−1m−1)yi−m,y1−m=y2−m=⋯=y0=0,

for sufficiently large *k*; for instance, *k* > 2*m*. We use this fact in the following examples.

Recently, the difference sequence spaces have been studied in [10, 34, 36–39].

Let *X* be nonempty set. Then a family of sets *I* ⊂ 2^*X*^ (power sets of *X*) is said to be an *ideal* if *I* is additive, that is, *A*, *B* ∈ *I* implies *A* ∪ *B* ∈ *I*, and hereditary; that is, *A* ∈ *I*, *B* ⊂ *A* implies *B* ∈ *I*.

A nonempty family of sets *F* ⊂ 2^*X*^ is said to be a *filter* of *X* if and only if (i) *ϕ* ∉ *F*, (ii) *A*, *B* ∈ *F* implies *A*∩*B* ∈ *F*, and (iii) *A* ∈ *F*, *A* ⊂ *B* implies *B* ∈ *F*.

An ideal *I* ⊂ 2^*X*^ is called *nontrivial* if *I* ≠ 2^*X*^.

A nontrivial ideal *I* is said to be *admissible* if *I*⊃{{*x*} : *x* ∈ *X*}.

If *I* is a nontrivial ideal in *X*(*X* ≠ *ϕ*), then the family of sets *F*(*I*) = {*M* ⊂ *X* : (∃*A* ∈ *I*)(*M* = *X*∖*A*)} is a filter of *X*, called the* filter associated with I*.

Throughout the paper *I* will stand for a nontrivial admissible ideal of *ℕ*.

We now introduce our main definitions.

**Definition 1 (see [40])**

A sequence *x* ∈ *w* is said to be *I*-convergent if there exists *L* ∈ *ℂ* such that, for all *ε* > 0, the set {*n* ∈ *ℕ* : |Δ^*m*^ *x*_*k*_ − *L*| ≥ *ε*} ∈ *I*. In this case, one writes (Δ^*m*^, *I*) − lim⁡*x*_*k*_ = *L*. The set of all (Δ^*m*^, *I*)-convergent sequences will be denoted by *c*(Δ^*m*^, *I*).

**Definition 2**

Let *α* ∈ (0,1] be any real number. The sequence *x* ∈ *w* is said to be (Δ^*m*^, *I*)-statistical convergence of order *α* (or *S* ^*α*^(Δ^*m*^, *I*)-convergence) if there is a real number *L* such that

(5) $$\begin{array}{c} \left\{n\in \mathbb{N}:\frac{1}{n^{\alpha }}\left|\left\{k\leq n:\left|\Delta^{m}x_{k}-L\right|\geq \varepsilon \right\}\right|\geq \delta \right\}\in I. \end{array}$$

In this case, we write *S* ^*α*^(Δ^*m*^, *I*) − lim⁡*x*_*k*_ = *L* or *S* ^*α*^(*I*) − lim⁡Δ^*m*^ *x*_*k*_ = *L*. The set of all (Δ^*m*^, *I*)-statistically convergent sequences of order *α* will be denoted by *S* ^*α*^(Δ^*m*^, *I*). In the special case *α* = 1, we will write *S*(Δ^*m*^, *I*) instead of *S* ^*α*^(Δ^*m*^, *I*).

(Δ^*m*^, *I*)-statistical convergence of order *α* is well defined for 0 < *α* ≤ 1, but it is not well defined for *α* > 1 in general. For this *x* = (*x*_*k*_) is defined as follows:

(6) $$\begin{array}{c} \Delta^{m}x_{k}=\{\begin{array}{cc} 1, & \text{if}\;k=2n;\\ 0, & \text{if}\;k\neq 2n; \end{array}\;n=1,2,3,\ldots . \end{array}$$

For every *ε* > 0 and *α* > 1 we have

(7) $$\begin{array}{c} \frac{1}{n^{\alpha }}\left|\left\{k\leq n:\left|\Delta^{m}x_{k}-1\right|\geq \varepsilon \right\}\right|\leq \frac{n}{2n^{\alpha }}, \end{array}$$

and so for any *δ* > 0

(8) {n∈ℕ:1nα|{k≤n:|Δmxk−1|≥ε}|≥δ} ⊂{n∈ℕ:n2nα≥δ}∈I,1nα|{k≤n:|Δmxk−0|≥ε}|≤n2nα,

we have

(9) {n∈ℕ:1nα|{k∈In:|Δmxk−0|≥ε}|≥δ} ⊂{n∈ℕ:n2nα≥δ}∈I.

Therefore, the sequence *x* = (*x*_*k*_) is (Δ^*m*^, *I*)-statistically convergent of order *α*, both to 1 and 0; that is, *S* ^*α*^(Δ^*m*^, *I*) − lim⁡*x*_*k*_ = 1 and *S* ^*α*^(Δ^*m*^, *I*) − lim⁡*x*_*k*_ = 0. But this is impossible.

It is easy to see that every (Δ^*m*^, *I*)-convergent sequence is (Δ^*m*^, *I*)-statistically convergent of order *α*(0 < *α* ≤ 1), but converse does not hold. For this, consider a sequence *x* = (*x*_*k*_) defined by

(10) $$\begin{array}{c} \Delta^{m}x_{k}=\{\begin{array}{cc} \frac{1}{\sqrt{k}}, & k\neq m^{3}\\ 1, & k=m^{3} \end{array}\;m=1,2,\ldots . \end{array}$$

It is clear that *x* ∈ *S* ^*α*^(Δ^*m*^, *I*) for *α* ∈ (1/3,1], but *x* ∉ *c*(Δ^*m*^, *I*).

**Definition 3**

Let *α* ∈ (0,1] be any real number and let *p* be a positive real number. A sequence *x* ∈ *w* is said to be strong (Δ_*p*_^*m*^, *I*)-Cesàro summable of order *α* (or strong *w* ^*α*^(Δ_*p*_^*m*^, *I*)-summable) if there is a real number *L* such that

(11) $$\begin{array}{c} \left\{n\in \mathbb{N}:\frac{1}{n^{\alpha }}\sum_{k=1}^{n}{\left|\Delta^{m}x_{k}-L\right|}^{p}\geq \varepsilon \right\}\in I. \end{array}$$

In this case, we write *w* ^*α*^(Δ_*p*_^*m*^, *I*) − lim⁡*x*_*k*_ = *L*. The set of all strong (Δ_*p*_^*m*^, *I*)-Cesàro summable sequences of order *α* to *L* will be denoted by *w* ^*α*^(Δ_*p*_^*m*^, *I*). In the special case *α* = 1, we will write *w*(Δ_*p*_^*m*^, *I*) instead of *w* ^*α*^(Δ_*p*_^*m*^, *I*).

## 3. Main Results

In this section, we give the main results of this paper. In Theorem 8 we give the inclusion relations between the sets of *S* ^*α*^(Δ^*m*^, *I*)-convergent sequences and *w* ^*α*^(Δ_*p*_^*m*^, *I*)-summable sequences. In Theorem 10, we give the relationship between *w* ^*α*^(Δ_*p*_^*m*^, *I*)-summable sequences for different *α*'s. In Theorem 12, we give the relationship between the sets of *S* ^*α*^(Δ^*m*^, *I*)- convergent sequences for different *α*'s.

**Theorem 4**

Let *α* ∈ (0,1] be any real number and suppose that *S* ^*α*^(Δ^*m*^, *I*) − lim⁡*x*_*k*_ = *L*_1_, *S* ^*α*^(Δ^*m*^, *I*)  lim⁡*y*_*k*_ = *L*_2_, and *c* ∈ ℝ; then

- *S* ^*α*^(Δ^*m*^, *I*) − lim⁡*cx*_*k*_ = *cL*_1_,
- *S* ^*α*^(Δ^*m*^, *I*) − lim⁡(*x*_*k*_ + *y*_*k*_) = *L*_1_ + *L*_2_.

**Proof**

(i) Suppose that *S* ^*α*^(Δ^*m*^, *I*) − lim⁡*x*_*k*_ = *L*_1_ and *c* ∈ ℝ; then

(12) 1nα|{k≤n:|Δm(cxk)−cL1|≥ε}| ≤1nα|{k≤n:|Δmxk−L1|≥ε|c|}|<δ2,

and so *S* ^*α*^(Δ^*m*^, *I*) − lim⁡(*cx*_*k*_) = *cL*_1_.

(ii) Now suppose that *S* ^*α*^(Δ^*m*^, *I*) − lim⁡*x*_*k*_ = *L*_1_ and *S* ^*α*^(Δ^*m*^, *I*) − lim⁡*y*_*k*_ = *L*_2_; then we have

(13) A1={n∈ℕ:1nα|{k≤n:|Δmxk−L1|≥ε2}|<δ2}∈F(I),A2={n∈ℕ:1nα|{k≤n:|Δmyk−L2|≥ε2}|<δ2}∈F(I),

and so *A*_1_∩*A*_2_ ≠ *ϕ*. Now for all *n* ∈ *A*_1_∩*A*_2_, we have

(14) 1nα|{k≤n:|Δm(xk+yk)−(L1+L2)|≥ε}| ≤1nα|{k≤n:|Δmxk−L1|≥ε2}|  +1nα|{k≤n:|Δmyk−L2|≥ε2}|<δ.

Then

(15) A3={n∈ℕ:1nα|{k≤n:|Δm(xk+yk)−(L1+L2)|≥ε}|<δ}∈F(I).

Hence *S* ^*α*^(Δ^*m*^, *I*) − lim⁡(*x*_*k*_ + *y*_*k*_) = *L*_1_ + *L*_2_.

The proofs of the following two theorems are easy and thus omitted.

**Theorem 5**

Let *α* ∈ (0,1] be any real number; then the limit of any *S* ^*α*^(Δ^*m*^, *I*)-convergent sequence is uniquely determined.

**Theorem 6**

Let *x* = (*x*_*k*_), *y* = (*y*_*k*_), and *z* = (*z*_*k*_) be real sequences such that Δ^*m*^ *x*_*k*_ ≤ Δ^*m*^ *y*_*k*_ ≤ Δ^*m*^ *z*_*k*_. If *S* ^*α*^(Δ^*m*^, *I*) − lim⁡*x*_*k*_ = *L* = *S* ^*α*^(Δ^*m*^, *I*) − lim⁡*z*_*k*_, then *S* ^*α*^(Δ^*m*^, *I*) − lim⁡*y*_*k*_ = *L*.

The proof of the following theorem is obtained by using the same techniques of Savas and Das [21, Theorem 2.4]; therefore we give it without proof.

**Theorem 7**

Let *α* ∈ (0,1] be any real number, then *S* ^*α*^(Δ^*m*^, *I*)∩*ℓ*_*∞*_(Δ^*m*^) is a closed subset of *ℓ*_*∞*_(Δ^*m*^).

In the following theorem we investigate the relationship between *S* ^*α*^(Δ^*m*^, *I*)-statistically convergent sequences and strong *w* ^*α*^(Δ_*p*_^*m*^, *I*)-summable sequences.

**Theorem 8**

Let *α* and *β* be fixed real numbers such that 0 < *α* ≤ *β* ≤ 1, and let *p* be a positive real number; then *w* ^*α*^(Δ_*p*_^*m*^, *I*) ⊂ *S* ^*β*^(Δ^*m*^, *I*), and the inclusion is strict.

**Proof**

Let *ε* > 0 and *w* ^*α*^(Δ_*p*_^*m*^, *I*) − lim⁡*x*_*k*_ = *L*; then we can write

(16) ∑k=1n|Δmxk−L|p≥∑k=1, ∣ Δmxk−L ∣ ≥εn|Δmxk−L|p≥εp|{k≤n:|Δmxk−L|≥ε}|,

and so

(17) $$\begin{array}{c} \frac{1}{\varepsilon^{p}n^{\alpha }}\sum_{k=1}^{n}{\left|\Delta^{m}x_{k}-L\right|}^{p}\geq \frac{1}{n^{\beta }}\left|\left\{k\leq n:\left|\Delta^{m}x_{k}-L\right|\geq \varepsilon \right\}\right|. \end{array}$$

Then for any *δ* > 0, we have

(18) {n∈ℕ:1nα|{k≤n:|Δmxk−L|≥ε}|≥δ} ⊆{n∈ℕ:1nβ∑k=1n|Δmxk−L|p≥εpδ}∈I.

This completes the proof.

Taking *α* = *β*, we show the strictness of the inclusion *w* ^*α*^(Δ_*p*_^*m*^, *I*) ⊂ *S* ^*β*^(Δ^*m*^, *I*) for a special case. For this, consider the sequence *x* = (*x*_*k*_) defined by

(19) $$\begin{array}{c} \Delta^{m}x_{k}=\{\begin{array}{cc} 1, & \text{if}\;k=m^{2},\\ 0, & \text{if}\;k\neq m^{2}, \end{array}\;m=1,2,\ldots . \end{array}$$

For every *ε* > 0 and *α* ∈ (1/2,1] we have

(20) $$\begin{array}{c} \frac{1}{n^{\alpha }}\left|\left\{k\leq n:\left|\Delta^{m}x_{k}-0\right|\geq \varepsilon \right\}\right|\leq \frac{\sqrt{n}}{n^{\alpha }}=\frac{1}{n^{\alpha -1/2}}, \end{array}$$

and for any *δ* > 0 we get

(21) {n∈ℕ:1nα|{k≤n:|Δmxk−0|≥ε}|≥δ} ⊂{n∈ℕ:[n]nα≥δ}.

Since the set on the right-hand side is a finite set and so belongs to *I*, it follows that *x*_*k*_ → 0(*S* ^*α*^(Δ^*m*^, *I*)) for *α* ∈ (1/2,1]. On the other hand, for *α* ∈ (0,1/2] we have

(22) $$\begin{array}{c} \frac{\sqrt{n}-1}{n^{\alpha }}\leq \frac{1}{n^{\alpha }}\sum_{k=1}^{n}{\left|\Delta^{m}x_{k}\right|}^{p}=\frac{1}{n^{\alpha }}\sum_{k=1}^{n}{\left|\Delta^{m}x_{k}-0\right|}^{p}. \end{array}$$

Then

(23) {n0,n0+1,n0+2,…}={n∈ℕ:n−1nα≥1}⊂{n∈ℕ:1nα∑k=1n|Δmxk−0|≥1},

for some *n*_0_ ∈ *ℕ* which belongs to *F*(*I*), since *I* is admissible. So *x*_*k*_↛*w* ^*α*^(Δ_*p*_^*m*^, *I*).

The converse of Theorem 8 does not hold, in general. To show this, we must find a sequence that is Δ^*m*^-bounded and *S* ^*α*^(Δ^*m*^, *I*)-convergent, but need not to be *w* ^*α*^(Δ_*p*_^*m*^, *I*)-summable. For this, consider a sequence *x* = (*x*_*k*_) defined by (10). It can be shown that *x* ∈ *ℓ*_*∞*_(Δ^*m*^) and *x* ∈ *S* ^*α*^(Δ^*m*^, *I*) for *α* ∈ (1/3,1] and *x* ∉ *w* ^*α*^(Δ_*p*_^*m*^, *I*) for *α* ∈ (0,1/2). Therefore, *x* ∈ *S* ^*α*^(Δ^*m*^, *I*)∖*w* ^*α*^(Δ_*p*_^*m*^, *I*) for *α* ∈ (1/3, 1/2).

The following result is a consequence of Theorem 8.

**Corollary 9**

If a sequence is *w* ^*α*^(Δ_*p*_^*m*^, *I*)-convergent to *L*, then it is *S* ^*α*^(Δ^*m*^, *I*)-convergent to *L*.

**Theorem 10**

Let 0 < *α* ≤ *β* ≤ 1, and let *p* be a positive real number; then *w* ^*α*^(Δ_*p*_^*m*^, *I*) ⊂ *w* ^*β*^(Δ_*p*_^*m*^, *I*) and the inclusion is strict.

**Proof**

The inclusion part of proof is trivial. Taking *p* = 1, we show the strictness of the inclusion *w* ^*α*^(Δ_*p*_^*m*^, *I*) ⊂ *w* ^*β*^(Δ_*p*_^*m*^, *I*) for a special case. Define the sequence *x* = (*x*_*k*_) such that

(24) $$\begin{array}{c} \Delta^{m}x_{k}=\{\begin{array}{cc} 1, & \text{if}\;k=m^{2},\\ 0, & \text{if}\;k\neq m^{2}, \end{array}\;m=1,2,\ldots . \end{array}$$

It can easily be shown that

(25) 1nβ∑k=1n|Δmxk−0|≤nnβ=1nβ−1/2⟶0,(n⟶∞)  for  β∈(12,1),

but

(26) 1nα∑k=1n|Δmxk−0|=1nα∑k=1n|Δmxk−0|≥n−1nα⟶∞,(n⟶∞)for  α∈(0,12).

So *x* ∈ *w* ^*β*^(Δ_*p*_^*m*^, *I*) for 1/2 < *β* < 1 but *x* ∉ *w* ^*α*^(Δ_*p*_^*m*^, *I*) for 0 < *α* < 1/2.

The following result is a consequence of Theorem 10.

**Corollary 11**

Let 0 < *α* ≤ *β* ≤ 1 be a positive real number. Then

- if *α* = *β*, then *w* ^*α*^(Δ_*p*_^*m*^, *I*) = *w* ^*β*^(Δ_*p*_^*m*^, *I*),
- *w* ^*α*^(Δ_*p*_^*m*^, *I*) ⊂ *w*(Δ_*p*_^*m*^, *I*) for each *α* ∈ (0,1].

**Theorem 12**

Let *α* and *β* be fixed real numbers such that 0 < *α* ≤ *β* ≤ 1; then *S* ^*α*^(Δ^*m*^, *I*) ⊂ *S* ^*β*^(Δ^*m*^, *I*), and the inclusion is strict.

**Proof**

Let *x* ∈ *S* ^*α*^(Δ^*m*^, *I*). Then given *α* and *β* such that 0 < *α* ≤ *β* ≤ 1, we may write

(27) 1nβ|{k≤n:|Δmxk−L|≥ε}| ≤1nα|{k≤n:|Δmxk−L|≥ε}|,{n∈ℕ:1nβ|{k≤n:|Δmxk−L|≥ε}|≥δ} ⊆{n∈ℕ:1nα|{k≤n:|Δmxk−L|≥ε}|≥δ}∈I

and this gives that *S* ^*α*^(Δ^*m*^, *I*) ⊂ *S* ^*β*^(Δ^*m*^, *I*).

We show the strictness of the inclusion *S* ^*α*^(Δ^*m*^, *I*) ⊂ *S* ^*β*^(Δ^*m*^, *I*) for a special case. Define the sequence *x* = (*x*_*k*_) such that

(28) $$\begin{array}{c} \Delta^{m}x_{k}=\{\begin{array}{cc} k, & \text{if}\;k=m^{2},\\ 0, & \text{if}\;k\neq m^{2}, \end{array}\;m=1,2,\ldots . \end{array}$$

Then *x* ∈ *S* ^*β*^(Δ^*m*^, *I*) for 1/2 < *β* ≤ 1, but *x* ∉ *S* ^*α*^(Δ^*m*^, *I*) for 0 < *α* ≤ 1/2.

The following result is a consequence of Theorem 10.

**Corollary 13**

Let 0 < *α* ≤ 1 be a real number; then *S* ^*α*^(Δ^*m*^, *I*) ⊂ *S*(Δ^*m*^, *I*).
